# The effect of transmission variance on observer placement for source-localization

**DOI:** 10.1007/s41109-017-0040-5

**Published:** 2017-07-11

**Authors:** Brunella Spinelli, L. Elisa Celis, Patrick Thiran

**Affiliations:** École Polytechnique Fédérale de Lausanne (EPFL), Lausanne, Switzerland

**Keywords:** Source localization, Epidemics, Sensor placement

## Abstract

Detecting where an epidemic started, i.e., which node in a network was the source, is of crucial importance in many contexts. However, finding the source of an epidemic can be challenging, especially because the information available is often sparse and noisy. We consider a setting in which we want to localize the source based exclusively on the information provided by a small number of *observers* – i.e., nodes that can reveal if and when they are infected – and we study where such observers should be placed.

We show that the optimal observer placement depends not only on the topology of the network, but also on the variance of the node-to-node transmission delays. We consider both low-variance and high-variance regimes for the transmission delays and propose algorithms for observer placement in both cases. In the low-variance regime, it suffices to only consider the network-topology and to choose observers that, based on their distances to all other nodes in the network, can distinguish among possible sources. However, the high-variance regime requires a new approach in order to guarantee that the observed infection times are sufficiently informative about the location of the source and do not get masked by the noise in the transmission delays; this is accomplished by additionally ensuring that the observers are not placed too far apart.

We validate our approaches with simulations on three real-world networks. Compared to state-of-the-art strategies for observer placement, our methods have a better performance in terms of source-localization accuracy for both the low- and the high-variance regimes.

## Introduction

**Fig. 1:**
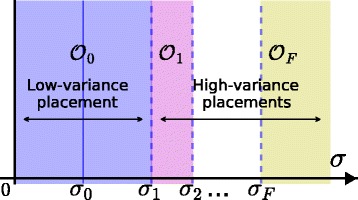
Sequence of optimal observer placements for increasing transmission variance. We assume the transmission transmission delays {*X*_*uv*_}_*uv*∈*E*_ to be such that $\mathbf {E}[\!X_{uv}] = w_{uv} \in \mathbb {R}_{+}$ and such that the variance is a growing function of a variance parameter *σ*, i.e., Var(*X*_*uv*_)=*g*(*w*_*uv*_,*σ*) with *g*(*x*,0)=0 for all $x\in \mathbb {R}^{+}$. For *σ*∈(0,*σ*_0_) the transmission delays are effectively deterministic (i.e., *σ* does not affect source localization). For *σ*∈(*σ*_0_,*σ*_1_), *σ* affects the accuracy of source localization but the optimal observer placement is still $\mathcal {O}_{0}$. For larger *σ*, the optimal observer placement might change, possibly multiple times ($\mathcal {O}_{k}$ denotes the optimal placement for *σ*∈(*σ*_*k*_,*σ*_*k*+1_)) up to *σ*=*σ*_*F*_. For *σ*>*σ*_*F*_ the optimal placement remains the same ($\mathcal {O}_{F}$)

**Fig. 2:**
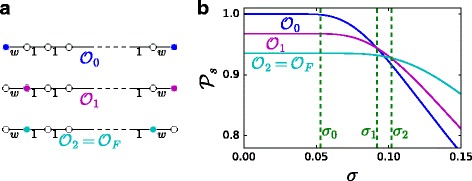
Optimal observers for Gaussian-distributed transmission delays with unit mean and standard deviation *σ* on a path graph. In this case $\mathcal {P}_{s}$ and, consequently, the optimal observer placements, can be explicitly computed. **a** different observer placements; **b** their performance in terms of probability of success $\mathcal {P}_{s}$ for *w*=20 and 30 edges

Regardless of whether a network comprises computers, individuals or cities, in many applications we want to detect whenever any anomalous or malicious activity spreads across the network and, in particular, where the activity originated. In effect, we wish to answer questions such as *what was the origin of a worm in a computer network?*, *who was the instigator of a false rumor in a social network?* and *can we identify patient zero of a virulent disease?* We call the spread of any such phenomenon an *epidemic* and its originator the *source*. Clearly, monitoring all network nodes is not feasible due to cost and overhead constraints: The number of nodes in the network may be prohibitively large and some of them may be unable or unwilling to provide information about their state. Thus, studies have focused on how to localize the source based on information from a few nodes (called *observers*). Given a set of observers, many models and estimators for source localization have been developed (Pinto et al. 2012; Louni and Subbalakshmi 2014; Zhang et al. 2016). However, the *selection* of observers has not yet received a satisfactory answer: Most methods consider only the structure of the network when placing observers. However, depending on the particular epidemic model, the expected transmission delay between two nodes, and its variance, can differ widely and this can have a significant impact on source localization. We show that different transmission models require different observer placements as illustrated in Figs. 1 and 2: As the variance of the transmission delays changes, the optimal set of observers also changes.

The difficulties faced in finding the optimal observers for source localization are two-fold. First, computing the likelihood of a node being the source conditional on the available observations can be computationally prohibitive (Shah and Zaman 2011; Pinto et al. 2012); evaluating the probability of correct localization given a set of observers is, in general, even harder. Second, the optimal selection of a limited number of observers is NP-hard, even when the transmission delays are deterministic. We take a principled approach that begins with considering deterministic transmission delays (*zero-variance* regime), and we build on this intuition in order to develop heuristics for both *low-variance* and *high-variance* regimes for the transmission delays.^1^

### Model and problem statement

**Transmission model.** We assume that the epidemic spreads in a known contact network. The *transmission delay* through edge *uv*, i.e., the time it takes for a node *u* to infect a neighbor node *v* is encoded by the random variable *X*_*uv*_.

We assume a transmission model which is both natural and versatile as it comprises deterministic transmissions, which we call *zero-variance*, and arbitrary *random* independent transmission models. We study, in particular, how the *amount* of randomness (i.e., the variance of *X*_*uv*_) in the transmission delays affects the choice of observers for source localization. Towards this, we are the first to separately analyze two different regimes for the amount of randomness of the transmission delays: *low-variance* and *high-variance*. A dichotomy exists between the two, and our approach for observer placement differs.

We use the SI epidemic model adopted, e.g., in (Pinto et al. 2012; Luo and Tay 2012). Nonetheless, since our methods for source localization only uses the time at which the sensors are first infected (no assumption on recovery or re-infection dynamics is made), they can be applied to any epidemic model, including the well known SIS or SIR (provided that nodes do not recover before infecting their neighbors).

**Source localization.** We assume that there is a *single* source that initiates the epidemic, an extension of our results to the case the case of multiple sources could use the recent work by Zhang et al. (2015) on a related problem and is left for future work.

Let $\mathcal {O} \subseteq V$ be the set of observer nodes (which we will select). We assume we know the time at which each observer is infected, and we refer to this vector of infection times as $T_{\mathcal {O}}$. Knowing $T_{\mathcal {O}}$ is a standard and realistic assumption (Netrapalli and Sanghavi 2012). We want to identify the source using only the information contained in $T_{\mathcal {O}}$.

We use maximum likelihood estimation (MLE) to produce an estimate $\hat s$ of the true unknown source *s* ^⋆^ as in (Pinto et al. 2012). This approach is common (see e.g., (Shah and Zaman 2011; Dong et al. 2013)), although the exact form of the estimator depends on the model and assumptions. In our case we have

$$\hat{s}\in \text{argmax}_{\substack{s \in V}} \mathbf{P}(T_{\mathcal{O}}|s^{\star}=s)\pi(s^{\star}), $$

where *π* denotes the prior on the position of the source. In this paper, unless otherwise specified we assume *π* to be uniform (i.e., *π*(*s* ^⋆^)=1/*n* for all nodes *s*∈*V* where *n*=|*V*|).

**Metrics.** We assume that we are given a *budget* *k* on the number of observers we can use, and that we must select our observers *once and for all*, i.e., independently of any particular epidemic instance. In order to select the *best set of observers* $\mathcal {O}$ *of size k* we must first define our metric of interest. In this work we are mainly interested in the *success probability*

$$\mathcal{P}_{s}=\mathbf{P}(\hat{s} = {s^{\star}}) $$

which is a widely used metric for source localization (see, e.g., (Shah and Zaman 2011; Pinto et al. 2012; Louni and Subbalakshmi 2014)). In our experiments we also evaluate another important metric, the *expected distance* between the estimated source and the real source (Celis et al. 2015; Louni et al. 2015), i.e., $\mathbf {E}[\!d(s^{\star },\hat {s})]$, where *d* denotes the distance between two nodes in the network.

In “Metrics for source localization” section we present several alternatives to these two metrics, including worst-case metrics, and show that optimizing different metrics can require different sets of observers.

### Main contributions

**Low-variance regime.** When the variance in the transmission delays is *low* (see “The low-variance regime” section), we prove that the set of optimal observers is exactly the optimal set for the zero-variance regime. In the zero- and low- variance regime, both the probability of success $\mathcal {P}_{s}$ (as well as other possible metrics of interest) can be explicitly computed. Despite this seeming simplicity, the problem remains NP-hard. We tackle the problem by using its connection with the well-studied related Double Resolving Set (DRS) problem (Cáceres et al. 2007) that minimizes the number of observers for correct localization. This minimum number is, in many cases, still prohibitively large, and can be as much as *n*−1, hence we cannot use this approach directly. However, from the connection between observer placement and DRS, we find inspiration for our algorithm which, by selecting one observer at a time until the budget is exhausted in order to reach a DRS set, greedily improves $\mathcal {P}_{s}$.

**High-variance regime.** When the noise in the transmission delays is *high*, it is no longer negligible and it poses an additional challenge to source localization; in effect, the accumulation of noise from node to node as the epidemic spreads might no longer enable us to distinguish between two potential sources, especially when they are both *far* from all observers. Hence, we must *strengthen* the requirements for observer placement in order to ensure that the nodes can be distinguished by observers that are *near* to them; this nearness is a function of the noise, of the budget *k*, and of the network topology. We define a novel objective function that both maximizes the success probability and imposes a *uniform* spread of observers in the network. Taking inspiration from the low-variance regime, we design an algorithm that greedily maximizes this new objective (see “The high-variance regime” section).

**Empirical results.** In “Empirical results” section, we evaluate our algorithms on three different real-world datasets that represent different application areas for source localization and different network topologies. First, we take a community of people living in the proximity of a university campus (Aharony et al. 2011), a typical network for the transmission of airborne diseases. Second, we take a community of students exchanging messages over a Facebook-like social network (Opsahl and Panzarasa 2009) through which ideas and trends can propagate. Finally, we consider the road network of the state of California (California Road Network): this captures geographical networks that can model the transmission of a disease between connected communities or the diffusion of contaminants, e.g., through a hydrological network. We show that our methods perform favourably against state-of-the-art approaches in both the low- and the high-variance regimes (see “Comparison against benchmarks” section). For the low-variance regime, we further compare our method against two other natural greedy heuristics for observer placement (see “Comparison with benchmarks” section); we show that our approach outperforms the rest. Moreover, in the empirical results, the dichotomy between the low- and high-variance regimes becomes apparent.

## Preliminaries

### Model

Let $\mathcal {G} = (V, E, w)$ be a weighted network. For ease of presentation we assume the graph is undirected and *w*_*uv*_=*w*_*vu*_; however our definitions and approach extend straightforwardly to the directed case. Assuming *u* is infected, the weight $w_{uv}\in \mathbb {R}_{+}$ of edge *u* *v*∈*E* represents the expected time it takes for *u* to infect *v*. The edge weights induce a *weighted-distance* metric *d* on $\mathcal {G}$: *d*(*u*,*v*) is the length of the shortest path from *u* to *v*. We also sometimes consider the minimum number of edges on a path connecting two nodes, which we call the *hops-distance*.

We assume that the epidemic is initiated by a single unknown source *s* ^⋆^ at an unknown time *t* ^⋆^. The fact that the *time* *t* ^⋆^ at which an epidemic starts is unknown adds a significant difficulty to the problem because a *single* observation is not *per se* informative. Instead, in order to localize the source, we must use the *differences* between the observed infection times.

If a node *u* gets infected at time *t*_*u*_, a non-infected neighbor *v* of *u* will become infected at time *t*_*v*_=*t*_*u*_+*X*_*uv*_ where *X*_*uv*_ is a random variable. A large part of the epidemic literature models transmission delays with exponential random variables. However we make a different modeling choice for two reasons. First, we are interested in decoupling the transmission variance and the average transmission time (for exponential random variables, mean and variance cannot be tuned independently). Second, in many applications it has been suggested that the transmission delays can be less-skewed than exponential random variables (Cha et al. 2009; Lessler et al. 2009; Vergu et al. 2010). For every edge *uv* we assume *X*_*uv*_ to be a symmetric and non-negative^2^ random variable. We do not make any strong assumption on the distribution of the transmission delays *X*_*uv*_: we only assume that their mean is equal to the edge weights, i.e., **E**[*X*_*uv*_]=*w*_*uv*_ for every *u* *v*∈*E*, and that their variance is an increasing function of both the edge weight and of a variance parameter *σ*, that is, Var(*X*_*uv*_)=*g*(*w*_*uv*_,*σ*), where *g* depends on the particular distribution of *X*_*uv*_ and *g*(*x*,0)=0 for all $x\in \mathbb {R}^{+}$.

If the variance is zero, or if it is low compared to edge weights, network distances are a good proxy for time delays (see “Identification of the source class” section). We refer to this setting as a *low-variance* regime, as opposed to the *high-variance* regime in which time delays are very noisy and network distances no longer work as a proxy for time delays.

### Distance vectors and node equivalence

We start with a few definitions. Our setting is similar to that of Celis et al. (2015).

#### **Definition 1**

*(Equivalence)* Let $\mathcal {G}=(V,E)$ and $\mathcal {O} \subseteq V$ with $|\mathcal {O}|=k \geq 2$ be a set of observers on $\mathcal {G}$. A node *u* is said to be equivalent to a node *v* (which we write *u*∼*v*) if and only if, for every $o_{i}, o_{j} \in \mathcal {O}$

1 $$  d(u, o_{i}) -d(u, o_{j}) = d(v, o_{i}) - d(v, o_{j}).  $$

**Fig. 3:**
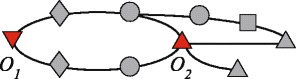
An unweighted network with two observer nodes *o*_1_ and *o*_2_. *Different shapes* represent different equivalence classes, i.e., groups of nodes which are not distinguishable from the point of view of the observers. In this example there are *q*=5 equivalence classes

The relation ∼ is reflexive, symmetric, and transitive, hence it defines an *equivalence relation*. Therefore, a set of observers $\mathcal {O}$ partitions *V* in *equivalence classes* (an example is given in Fig. 3). We denote by *q* the number of equivalence classes and we let $[u]_{\mathcal {O}}$ be the class of *u*, i.e., the set of all nodes that are equivalent to *u*.

When the variance is zero, given an observer set, we can *distinguish* *u* from *v* if there exist two observers *o*_*i*_,*o*_*j*_ such that Eq. (1) does *not* hold for *u*,*v* and *o*_*i*_,*o*_*j*_, i.e.,

$$d(u, o_{i}) -d(u, o_{j}) \neq d(v, o_{i}) - d(v, o_{j}), $$

which means that $[\!u]_{\mathcal {O}} \neq [\!v]_{\mathcal {O}}$.

The problem of finding the minimum-size set of nodes *S*, such that for every *u*,*v* in a network there exist *s*_*i*_,*s*_*j*_∈*S* for which *d*(*u*,*s*_*i*_)−*d*(*u*,*s*_*j*_)≠*d*(*v*,*s*_*i*_)−*d*(*v*,*s*_*j*_) is known as the *Double Resolving Set (DRS) Problem* (Cáceres et al. 2007), while the minimum size of a DRS is known as the *Double Metric Dimension* (DMD) of the network. Our problem differs from DRS because we focus on the more realistic context in which, due to limited resources, we want to allocate a *finite budget* in order to optimize source localization^3^ (as opposed to minimizing the number of observers for perfect localization, which is, in many cases, still prohibitively large). However, the connection between our problem and DRS paves the way for a principled approach to observer placement.

We now define, for every *v*∈*V*, a *distance vector*, which, as we will see in Lemma 1, mathematically captures equivalence in a manner that is easy to work with.

#### **Definition 2**

*(Distance Vector)* Let $\mathcal {G} = (V,E)$, $\mathcal {O}\subseteq {V}$ with $|\mathcal {O}|=k \geq 2$ and $o_{1}\in \mathcal {O}$. For each node *v*∈*V* the distance vector of *v* with respect to *o*_1_ is $\mathbf {d}_{s, o_{1}} \in \mathbb {R}^{k-1}$ with entries *d*(*v*,*o*_*i*+1_)−*d*(*v*,*o*_1_) for 1≤*i*≤*k*−1.

The following lemma, similar in spirit to Lemma 3.1 in (Chen et al. 2014), shows that the equality between distance vectors of different nodes does not depend on the choice of the *reference observer* *o*_1_.

#### **Lemma 1**

Let $\mathcal {G} = (V,E)$ and $\mathcal {O} \subseteq V$ with $|\mathcal {O}|=k \geq 2$ and let *u*,*v*∈*V*. Then, $[u]_{\mathcal {O}}$=$[v]_{\mathcal {O}}$ if and only if $\mathbf {d}_{u, o_{1}} = \mathbf {d}_{v, o_{1}}$, independently of the choice of the reference observer *o*_1_.

### Metrics for source localization

In this section we define some possible metrics of interest for the source-localization problem and we show that optimizing these metrics can effectively require different sets of observers.

For ease of exposition, we restrict ourselves to the zero-variance regime and we assume that the prior distribution on the position of the source is uniform.

In the zero-variance regime, the partition in equivalence classes is effectively the only factor for the localization of the source: if [*s* ^⋆^] is a singleton, it is always possible to localize the source exactly based on the observed infection time; if it is not a singleton, we can only correctly identify the *class* to which *s* ^⋆^ belongs and we produce an estimated source $\hat {s} \in [s^{\star }]$ sampling from [*s* ^⋆^] uniformly.

We adopt two metrics to evaluate the performance of our algorithms: the *success probability* $\mathcal P_{s}$ and the *expected error distance* $\mathcal {D}$.

The success probability $\mathcal {P}_{s}$ is defined as $\mathbf {P}(\hat {s} = s^{\star })$. In the low-variance case it can be easily computed. Let *q* be the number of equivalence classes identified by an observer set $\mathcal {O}$, then

2 $$ \begin{array}{ll} \mathcal{P}_{s} &=\sum\limits_{[u] \subseteq V}\mathbf{P}(\hat{s} = s^{\star}|s^{\star} \in\ [\!u])\mathbf{P}(s^{\star} \in\ [\!u])\\ &= \sum\limits_{[u]\subseteq V} \frac{1}{|[u]|} \cdot \frac{|[u]|}{n} = \frac 1 n \sum\limits_{[u] \subseteq V} 1 = \frac{q}{n}. \end{array}  $$

Note that $\mathcal {P}_{s} = 1$ if and only if all equivalence classes are singletons.

The expected error distance $\mathcal {D} \stackrel {\text {def}}{=} \mathbf {E}[\!d(\hat {s}, s^{\star })]$ can also be computed, in the low-variance case, from the partition in equivalence classes:

3 $$ \begin{array}{ll} \mathcal{D} &= \mathbf{E}[\!d(s^{\star}, \hat{s})] \\ &= \sum\limits_{s \in V}\mathbf{P}(s^{\star} = s)\sum\limits_{u \in [s]} \mathbf{P}(\hat s = u | s^{\star} = s) d(s,u) \\ &=\frac 1 n \sum\limits_{s \in V} \frac{1}{|[s]|}\sum\limits_{u \in [s]} d(s, u), \end{array}  $$

where again $\mathcal {D} = 0$ if and only if all equivalence classes are singletons. An analogous expression for the hops-distance (instead of the weighted distance as in (3)) is also considered in the experimental evaluation in “Empirical results” section.

Maximizing $\mathcal {P}_{s}$ (respectively, minimizing $\mathbf {E}[\!d(s^{\star }, \hat {s})]$) we minimize the probability of $\hat {s} \neq s^{\star }$ (respectively, the average distance between *s* ^⋆^ and $\hat {s}$). Other natural metrics of interest are the *worst-case* versions of these metrics over the vertex set *V*, i.e., the *minimum probability of success* $\widehat {\mathcal P_{s}}\stackrel {\text {def}}{=} \min _{[s] \subseteq V}\mathcal {P}_{s}|_{s^{\star } \in [s]}$ and the *maximum distance between* $\hat {s}$ *and* *s* ^⋆^, denoted by $\widehat {\mathcal {D}}$. $\widehat {\mathcal {P}_{s}}$ can be computed as

$$\widehat{\mathcal{P}_{s}} = \min_{[u]\subseteq V} \frac{1}{|[u]|}, $$

and $\widehat {\mathcal {D}}$ as

$$\widehat{\mathcal{D}} = \max_{[s] \subseteq V} \max_{t,v \in [s]} d(t,s). $$

These last two metrics are relevant, for example, in an adversarial setting (e.g., in the case of bio-warfare), where if the observers are known, the adversary would select the *worst* location for the source.

A last natural metric, which is intermediate between average and worst-case metrics, is the *expected maximum distance* between the true and the estimated source that we define as $\overline {\mathcal {D}} \stackrel {\text {def}}{=} \mathbf {E}_{s^{\star }}[\max (d(s^{\star }, \hat {s}))]$. We have

$$\overline{\mathcal{D}} = \mathbf{E}_{s^{\star}}[\max d(s^{\star}, \hat{s})] = \sum_{s \in V} \frac{1}{n} \left(\max_{t \in [s]} d(s,t)\right). $$

**Fig. 4:**
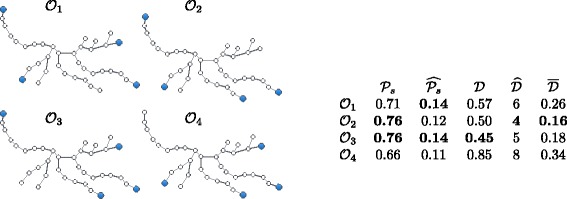
A tree with different sets of *k*=4 observers (*blue*). The table displays the values of different metrics for the observer sets. The best values for each metric are in *bold type*. The remaining possible choices of *k*=4 observers within the leaves set are omitted either because they are equivalent to one of the placements considered or because they do not optimize any of the metrics examined

We demonstrate an example which shows that optimizing these five metrics can require different set of observers. Consider the tree in Fig. 4 together with the four sets of *k*=4 observers represented in the four sub-figures. With an argument similar to that of Celis et al. (2015), it can be shown that, for all metrics considered and for any budget *k* smaller than the number of leaves, the optimal observer set is a subset of the leaves set.^4^ Hence we only consider observer sets contained in the leaves set. Figure 4 shows the values of $\mathcal {P}_{s}$, $\widehat {\mathcal P_{s}}$, $\mathcal {D}$, $\widehat {\mathcal {D}}$ and $\overline {\mathcal {D}}$ for a subset of the possible observer placements contained in the leaves set and having cardinality *k*=4. These placements include those that optimize $\mathcal {P}_{s}$, $\widehat {\mathcal {P}_{s}}$, $\mathcal {D}$, $\widehat {\mathcal {D}}$ and $\overline {\mathcal {D}}$.

**Fig. 5:**
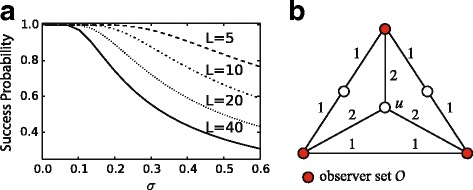
**a** Success probability $\mathcal {P}_{s}$ on a path of length *L* for increasing variance *σ*. **b** Counterexample for the converse of Lemma 2; for each pair of observers in $\mathcal {O}$, *u* is not contained in the shortest path between them, yet $\mathcal {O}$ is a DRS

## The low-variance regime

### Identification of the source class

We formalize how we can localize the source in the zero-variance setting, i.e., when *X*_*uv*_=*w*_*uv*_ for every edge (*u*,*v*).

For every observer $o_{i} \in \mathcal {O}$, denote by *t*_*i*_ the time at which *o*_*i*_ gets infected. In the zero-variance setting, the observed infection times of nodes *o*_2_,…,*o*_*K*_ with respect to observer *o*_1_, i.e., the vector ***τ***=def*t*_2_−*t*_1_,…,*t*_*k*_−*t*_1_, is exactly the distance vector of the *unknown* source *s* ^⋆^ with respect to *o*_1_. Then, if for every $u,v \in V [u]_{\mathcal {O}}\neq [v]_{\mathcal {O}}$, the source can be always correctly identified by finding the node whose distance vector matches the observed infection times. Theorem 1 proves that this is true also in a more general *low-variance* framework where we are always able to identify the equivalence class to which the real source belongs by looking at the distances between the distance vectors $\{\mathbf {d}_{v, o_{1}}, v \in V\}$ and the vectors of infection times ***τ***.

#### **Theorem 1**

Let $\mathcal {G}=(V,E)$ be a network of size *n*, $\mathcal {O}\subseteq {V}$ and fix $o_{1}\in \mathcal {O}$. Call

$$\delta\triangleq \min_{u, v: \mathbf{d}_{u, o_{1}} \neq \mathbf{d}_{v, o_{1}}}\|\mathbf{d}_{u, o_{1}}-\mathbf{d}_{v, o_{1}}\|_{\infty}$$

and call *D* the maximum distance in hops in any shortest path between any node and any observer.

If the transmission delays are such that for each *u* *v*∈*E*, *X*_*uv*_∈ [*w*_*uv*_(1−*ε*),*w*_*uv*_(1+*ε*)] with $\varepsilon <\varepsilon _{0} \triangleq \frac {\delta }{4D}$ then for every $v \in \ [s^{\star }] \|\mathbf {d}_{v, o_{1}} - \boldsymbol \tau \|_{\infty }\leq 2\varepsilon D$ and for every $v \notin \ [s^{\star }] \|\mathbf {d}_{v, o_{1}} - \boldsymbol \tau \|_{\infty } > 2\varepsilon D$.

#### *Proof*

Let $t_{o^{\prime }}$ be the infection time of ${o^{\prime }}\in \mathcal {O}$. When the source is *s* ^⋆^ we have

4 $$ t_{o^{\prime}} -t^{\star} \leq d(s^{\star}, {o^{\prime}})(1+\varepsilon).  $$

Moreover, if $\mathcal {Q}$ is the collection of all paths connecting *s* ^⋆^ and *o* ^′^ and, for $p \in \mathcal {Q}$, if *d*_*p*_(*s* ^⋆^,*o* ^′^) is the (weighted) length of path *p* we have

5 $$ t_{o^{\prime}} - t^{\star} \geq \min_{p\in \mathcal{Q}} d_{p}(s^{\star}, {o^{\prime}})(1-\varepsilon)=d(s^{\star}, {o^{\prime}})(1-\varepsilon).  $$

Combining inequalities (4) and (5) for *o* ^′^ being, respectively, *o* and *o*_1_ and calling *t*_1_ (resp., *t*_*o*_) the infection time of the reference observer *o*_1_ (resp., *o*), we have

$$\begin{array}{@{}rcl@{}} |t_{o} - t_{1} - d(s^{\star}, o) + d(s^{\star}, o_{1})|\leq\qquad\qquad&\\ &\qquad\varepsilon (d(s^{\star}, o) + d(s^{\star}, o_{1}))\leq 2\varepsilon D. \end{array} $$

Since for every $v \in [s^{\star }] \mathbf {d}_{v, o_{1}}=\mathbf {d}_{s^{\star }, o_{1}}$, we conclude that for every *v*∈ [*s* ^⋆^], $\|\mathbf {d}_{v, o_{1}} - \boldsymbol \tau \|_{\infty }\leq 2\varepsilon D$.

Take now *v*∉[*s* ^⋆^] and assume by contradiction that $\|\mathbf {d}_{v, o_{1}} - \boldsymbol \tau \|\leq 2\varepsilon D$. Using the triangular inequality and the hypothesis *ε*<*δ*/4*D* we have

$$\begin{array}{ll} \|\mathbf{d}_{s^{\star}, o_{1}} - \mathbf{d}_{v, o_{1}}\|_{\infty} &\leq \|\mathbf{d}_{s^{\star}, o_{1}} - \boldsymbol\tau\|_{\infty} + \|\mathbf{d}_{v, o_{1}} - \boldsymbol\tau\|_{\infty} \\ &\leq 4\varepsilon D < \delta, \end{array} $$

which contradicts the definition of *δ*. Hence for every *v*∉ [*s* ^⋆^], $\|\mathbf {d}_{v, o_{1}} - \boldsymbol \tau \|_{\infty } > 2\varepsilon D$. □

Note that here *ε*_0_ plays the role of *σ*_0_ in Fig. 1 in the sense that it is an upper-bound on a regime in which the delays are effectively deterministic and the variance of the transmission delays does not affect the accuracy of source localization.

If additional conditions on the weights or on the network topology are made, more refined versions of Theorem 1 can be proven. For example, in a *tree* with integer weights, due to the uniqueness of the path between two any vertices, it can be shown that *δ*≥2 and Theorem 1 holds for $\varepsilon <\varepsilon _{0} \triangleq \frac {1}{2D}$.

For the remainder of this section, we will assume *ε*<*δ*/4*D*, which we call the low-variance regime.

### Estimation of the source

Assume that a prior probability distribution on the identity of the source is given, i.e., that we know *π*(*v*)=def**P**(*s* ^⋆^=*v*). After the source class $[s^{\star }]_{\mathcal {O}}$ is identified based on ***τ*** as described in “Identification of the source class” section, we let our estimated source $\hat {s}$ be chosen at random from the conditional probability $\phantom {\dot {i}\!}\pi |_{[s^{\star }]}(u)\stackrel {\text {def}}{=}\mathbf {P}(s^{\star }=v|v \in [s^{\star }])$. If a prior *π* is not known, we select the estimated source uniformly at random from [*s* ^⋆^], which is equivalent to having a uniform prior *π*.

For ease of exposition, we focus on the case in which the prior distribution on the position of the source is uniform, hence *π*(*v*)=1/*n* for all *v*∈*V*. Our algorithms and observations can be easily extended to general priors.

### Observer placement

Independently of the topology of the network $\mathcal {G}$, the success probability $\mathcal {P}_{s}$, as well as other possible metrics of interest, can be computed exactly in polynomial time (see, e.g., Eqs. (2) and (3)). In fact, due to Lemma 1 and Theorem 1, it is enough to compute the distance vector of Definition 1 for all the nodes. Nonetheless, if we have a budget *k*≥2 of nodes that we can choose as observers, finding the configuration that maximizes $\mathcal {P}_{s}$ is an NP-hard problem. This is a direct consequence of the hardness result of Chen et al. (2014).

#### **Theorem 2**

Let *k*≥2 be the budget on the number of nodes we can select as observers. Finding $\mathcal {O} \subseteq V$ such that $\mathcal {O} \in \text {argmax}_{|\mathcal {O}|=k}\mathcal {P}_{s}(\mathcal {O})$ is NP-hard.

The proof follows straightforwardly with a reduction from the DRS problem (see Appendix B).

Our first main contribution in this paper is a solution to the budgeted observer-placement problem for general graphs.

For trees, the optimal observer placement can be find in polynomial time using dynamic programming techniques (Celis et al. 2015). In a general graph (with loops) the problem of source localization is made more challenging by the multiplicity of paths through which the epidemic can spread and for the same reason also finding an optimal observer set becomes much harder.

A first idea to solve observer placement on a general graph could be to use the latter result on a BFS-approximation of the graph. However, as mentioned in “Metrics for source localization” section, on a tree the optimal observer placement is contained in the leaves set. If we consider a non-tree graph and take a BFS-approximation, the leaves of the BFS tree depend on where the BFS-tree is rooted. Hence using the result of (Celis et al. 2015) on a tree approximation it is not possible to guarantee high probability of success independently of the position of the source.

Our approach, presented in Algorithm 1, does not rely on a graph approximation. Moreover, it is specifically designed for the source localization problem and has a simple greedy structure: for every node *v*∈*V*, initialize $\mathcal {O}\leftarrow \{v\}$ and iteratively add to $\mathcal {O}$ the node *u* that maximizes the gain with respect to the success probability until we either run out of budget or $\mathcal {P}_{s}=1$. Eq. 2 ensures that greedily maximizing the success probability is equivalent to greedily maximizing the number *q* of equivalence classes. When adding an element to the observer set, the partition in equivalence classes can be updated in linear time, total running time of our algorithm is *O*(*k* *n* ^3^). Despite bypassing the NP-hardness of the problem, this might not be sufficiently fast for very large networks. However, the procedure is extremely parallelizable (see, for example, the main for loop and the **a** **r** **g** **m** **a** **x** in the **w** **h** **i** **l** **e** loop).


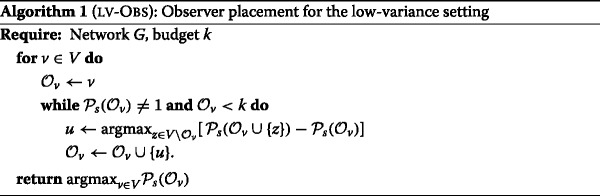


The osbserver placement obtained through Algorithm 1 will be denoted LV-OBS to emphasize the fact that it is designed for the case in which the variance is absent or very small (LV stands for *low-variance* regime).

**Fig. 6:**
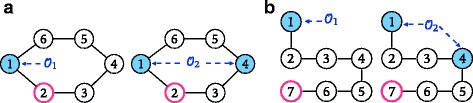
Counterexamples for the submodularity property of Algorithms 1 and 2. For the graph in (**a**) (respectively, (**b**)) the gain of adding the node with *red border* to $\mathcal {O}_{2}$ is larger in terms of $\mathcal P_{s}$ (respectively, *P*_*L*_) than the gain of adding it to $\mathcal {O}_{1} \subseteq \mathcal {O}_{2}$

Unfortunately we cannot use a submodularity argument to give guarantees on the performance of Algorithm 1 because the number of equivalent classes, and hence the function $\mathcal {P}_{s}$, are not submodular. Consider as a simple example a cycle of length 6 as in Fig. 6a. If the observer set is $\mathcal {O}_{1}=\{1\}$ the number of equivalence classes is *q*=1. If we add node 2 to $\mathcal {O}_{1}$ the classes become {1,5,6} and {2,3,4} (*q*=2). Hence by adding node 2 to the set {1} the gain in terms of equivalence classes is just 1. Consider now $\mathcal {O}_{2}=\{1, 4\}\supseteq \mathcal {O}_{1}$, which identifies as classes {1}, {4}, {2,6} and {3,5}. If again we add node 2 to $\mathcal {O}_{2}$ we reach a DRS of $\mathcal {C}$, i.e., all classes are singletons. This means that the gain in terms of equivalence classes is 6−4=2>1 and we conclude that the number of equivalence classes is not submodular.

### Comparison with benchmarks

As budgeted observer placement (even in the zero-variance setting) is NP-hard, there is no optimal algorithm to compare against. Instead, we evaluate the performance of our algorithm against a set of natural benchmarks that have shown to have good performance in other works (Seo et al. 2012; Berry et al. 2006; Zhang et al. 2016) (see “Comparison against benchmarks” section for a discussion of these benchmarks, Figs. 10-12 for the results).

**Alternative objective functions.** We further compare LV-OBS against two other natural heuristics that also optimize an objective function greedily.

The first is an adapted version of the approximation algorithm for the DRS problem proposed by Chen et al. (2014) and described in Appendix A.

By stopping the greedy process after it selects *k* nodes, we can adapt in a natural way this approximation algorithm and create a heuristic for the budgeted version that we denote by *Φ*_*ent*_. We want to check if LV-OBS actually reaches smaller values of $\mathcal {P}_{s}$ compared to *Φ*_*ent*_.

The second is a direct minimization of the expected error distance $\mathcal {D}= \mathbf {E}\, [\!d(s^{\star }, \hat {s})]$ of Eq. (3) that we denote by *Φ*_*dist*_. Even if LV-OBS is not directly minimizing $\mathcal {D}$, we want to compare the results we obtain in terms of $\mathcal {D}$ with those obtain to *Φ*_*dist*_ in order to check if, at least in some budget regimes, we can use the maximization of $\mathcal {P}_{s}$ as a proxy for the minimization of $\mathcal {D}$.

The results of our empirical evaluation are presented in Table 2 in Appendix C.

The results achieved by *Φ*_*ent*_ and *Φ*_*dist*_ are, on average, worse than those of Algorithm 1 both in terms of $\mathcal {P}_{s}$ and of $\mathcal {D}$, independently of the graph topology. We observe two exceptions. First, when *k* is very small: *Φ*_*dist*_ reaches smaller values of $\mathcal {D}$ compared to LV-OBS, which can explained by the fact that *Φ*_*dist*_ directly minimizes $\mathcal {D}$ and that, when fewer observers are available the difference between the observer placements that maximize $\mathcal {P}_{s}$ and minimize $\mathcal {D}$ is greater. Second, for large *k*, on the Barabàsi Albert networks *Φ*_*ent*_ gives, in average, larger $\mathcal {P}_{s}$ than LV-OBS. This is probably due to the fact that, for this class of graphs, the DMD is small, hence with a large value of *k* we approach the regime in which the objective function of *Φ*_*ent*_, designed to minimize the DMD of the network, is optimal.

## The high-variance regime

When the variance is not guaranteed to be low, as defined in “The low-variance regime” section, computing analytically the success probability - or other metrics of interest - is unfortunately not possible (except for very simple graphs, like the path network of Fig. 2, and for particular transmission delays, e.g., Gaussian-distributed).

When the variance is high, also the localization of the source is more challenging because the observed infection delays *t*_*i*_−*t*_*j*_ can be misleading, especially if the corresponding observers *o*_*i*_ and *o*_*j*_ are *far* from the source. Take, for example, a path of length *L* where the two leaves are the only two observers and all edges have weight equal to 1. Figure 5a shows how the success probability $\mathcal P_{s}$ decays faster for increasing values of *L*. Building on this observation, we propose a strategy for observer placement that enforces a controlled distance from a general source node to the observer set.

**Source localization.** For the high-variance case we localize the source using an adapted version of the algorithm proposed by Pinto et al. (2012) (see Appendix D for details). This adapted algorithm can be seen as a generalization to the high-variance regime of the source localization method presented in “Identification of the source class” section for the low-variance regime.

### Observer placement

First, we formalize why distances between observers are important. Recall that for every transmission delay *X*_*uv*_ we assume Var(*X*_*uv*_)=*g*(*w*_*uv*_,*σ*), with *g* being an increasing function of both its arguments. If *o*_*i*_,*o*_*j*_ are two observers connected by a unique path $\mathcal {P}(o_{i}, o_{j})$ and the source is ${v^{\star } \in \mathcal {P}(o_{i}, o_{j})}$, then

6 $$ \text{var}(t_{i} - t_{j}) = \left[\sum_{uv \in \mathcal{P}(o_{i}, o_{j})} g(w_{uv}, \sigma). \right].  $$

For example, if $X_{uv} \sim \mathcal {N}\left (w_{uv}, \sigma ^{2} w_{uv}^{2}\right)$ we have

7 $$ \text{var}(t_{i} - t_{j}) = \sigma^{2}\left[\sum_{uv \in \mathcal{P}(o_{i}, o_{j})} w_{uv}^{2} \right].  $$

Although we cannot control *σ*, we can control the *path length* between observers.

We make use of the following sufficient condition for a set to be a DRS, i.e., for an observer set to guarantee correct source localization.

#### **Lemma 2**

Let $\mathcal {G}=(V, E)$ be a network, $\mathcal {O} \subseteq V$. If for every *u*∈*V* there exist $o_{1}, o_{2} \in \mathcal {O}$ such that there is a unique shortest path $\mathcal {P}(o_{1}, o_{2})$ between *o*_1_ and *o*_2_ and $u \in \mathcal {P}(o_{1}, o_{2})$, then $\mathcal {O}$ is a DRS for *G*.

#### *Proof*

Let $u, v \in V \backslash \mathcal {O}$. We will prove that there exist $o_{1}, o_{2} \in \mathcal {O}$ such that the pair (*u*,*v*) is resolved by (*o*_1_,*o*_2_), i.e., *d*(*v*,*o*_1_)−*d*(*u*,*o*_1_)≠*d*(*v*,*o*_2_)−*d*(*u*,*o*_2_). Let $o_{1}, o_{2} \in \mathcal {O}$ such that *u* appears in the unique shortest path $\mathcal {P}(o_{1}, o_{2})$ and *o*_3_,*o*_4_∈*S* such that *v* appears in the unique shortest path $\mathcal {P}(o_{3}, o_{4})$. If $v \in \mathcal {P}(o_{1}, o_{2})$ or $u \in \mathcal {P}(o_{3}, o_{4})$ than *u* and *v* are resolved by, respectively, (*o*_1_,*o*_2_) or (*o*_3_,*o*_4_). Take $v \notin \mathcal {P}(o_{1}, o_{2})$ and $u \notin \mathcal {P}(o_{3}, o_{4})$. In this case, {*o*_1_,*o*_2_}≠{*o*_3_,*o*_4_}. Let us suppose without loss of generality that *o*_1_∉{*o*_3_,*o*_4_}. We look only at the case where (*o*_1_,*o*_2_) does not resolve (*u*,*v*) and prove that the pair is indeed resolved by two vertices in $\mathcal {O}$. Since (*o*_1_,*o*_2_) does not resolve (*u*,*v*), there exists $c\in \mathbb {R}$ such that *d*(*v*,*o*_1_)−*d*(*u*,*o*_1_)=*c*=*d*(*v*,*o*_2_)−*d*(*u*,*o*_2_). Since the unique shortest path between *o*_1_ and *o*_2_ goes through *u* we have that *c*>0. We prove that either (*o*_1_,*o*_3_) or (*o*_1_,*o*_4_) resolves (*u*,*v*). If this was not the case, we would have the following equalities:

$$\begin{array}{*{20}l} c &= d(v, o_{1})-d(u,o_{1}) = d(v, o_{3})- d(u, o_{3})\\ c &= d(v, o_{1})-d(u,o_{1}) = d(v, o_{4})- d(u, o_{4}). \end{array} $$

Since *c*>0, *d*(*v*,*o*_3_)>*d*(*u*,*o*_3_) and *d*(*v*,*o*_4_)>*d*(*u*,*o*_4_) giving a contradiction with *v* (and not *u*) being on the shortest path $\mathcal {P}(o_{3}, o_{4})$. We conclude that (*u*,*v*) are resolved by either (*o*_1_,*o*_3_) or (*o*_1_,*o*_4_). □

The converse of this lemma is not true: If $\mathcal {O}$ double resolves $\mathcal {G}$, it is not even true that for every node *u* there must exist $o_{1}, o_{2} \in \mathcal {O}$ such that *u* is contained in *some* shortest path between *o*_1_ and *o*_2_ of (see Fig. 5b).

**Path covering strategy.** We take Lemma 2 as a basis for deriving a *path covering* strategy for observer placement. In practice, the condition about the *uniqueness* of the shortest path is too strong and excludes many potentially useful observer nodes. Experimentally we see that in many practical situations two shortest paths differ only by a few nodes and the majority of nodes on the path are resolved by the two extreme nodes. This is why we relax the condition of Lemma 2 and we prefer, when the shortest path is not unique, to select one arbitrarily. Let *S*⊆*V* be a set of observers and *L* a positive integer: We call *P*_*L*_(*S*) the set of nodes that lie on a shortest path of length at most *L* between any two observers in the set *S*. Given a budget *k*, and a positive integer *L*, we denote by $S^{\star }_{k, L}$ the set of *k* vertices that maximize the cardinality of *P*_*L*_(*S*). We call *L* the *length constraint* for the observer placement because we consider an observer to be *useful* for source localization only if it is within distance *L* from another observer. $S^{\star }_{k, L}$ can be approximated greedily as in Algorithm 2. The running time of Algorithm 2 is *O*(*n* ^2^ *k* ^2^), however, as Algorithm 1, this algorithm is highly parallelizable and hence tractable even for large networks.


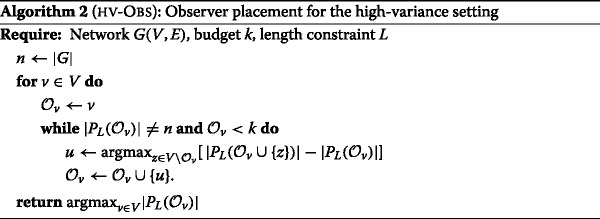


We will refer to the observer placement produced by Algorithm 2 as HV-OBS (*L*) to emphasize that it is designed for the high-variance case.

Unfortunately also for Algorithm 2 we cannot use a submodularity argument to derive approximation guarantees. In fact, the function *P*_*L*_ is not submodular. Consider the path $\mathcal {P}$ of 7 nodes in Fig. 6b, fix *L*=3 and set $\mathcal {O}_{1}=\{1\}$. If we add node 7 to $\mathcal {O}_{1}$ no node lies on a path of length smaller than *L*=3 among the two observers 1 and 7, hence the gain is 0. Consider now $\mathcal {O}_{2}=\{1, 4\}\supseteq \mathcal {O}_{1}$. If we add node 7 to $\mathcal {O}_{2}$, the gain is 3 because node 5,6 and 7, that did not lie on any path of length smaller than *L* connecting two observers before, now lie on the path connecting 4 and 7, hence *P*_*L*_ is not submodular.

**Comparison with Algorithm 1.** Note that taking *L* equal to the maximum weighted distance *Δ* between two nodes in $\mathcal {G}$ does not make Algorithm 2 equivalent to Algorithm 1, i.e., we do not obtain LV-OBS. To see how the two algorithms could give different results, take a cycle of odd length *d* with a leaf node *ℓ* added as a neighbor to an arbitrary node *v* and assume to start the algorithm with initial set {*v*}. At the first step, the two algorithms will make the same choice, choosing one of the two nodes that is at distance (*d*−1)/2 from *v*. At the second step however, LV-OBS will add *ℓ* (a DRS contains all leaves (Chen et al. 2014)), whereas Algorithm 2 will add a node on the cycle. This observation is key to our results because it explains why Algorithm 2 results in a more uniform (and hence *variance-resistant*) observer placement with respect to LV-OBS. HV-OBS operates a trade-off between the average distance to the observers and the maximization of $\mathcal {P}_{s}$.

**Choice of the L parameter.** How could one optimally set *L*? Needless to say, the optimal *L* depends on the network topology and on the available budget: Clearly, for a larger budget a smaller *L* is preferred.

**Fig. 7:**
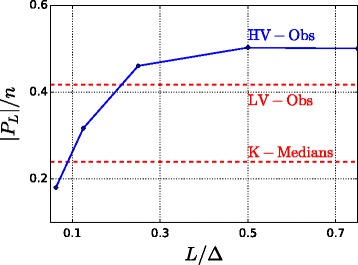
Fraction of nodes in *P*_*L*_(·) for the California dataset with 2% of observers

**Fig. 8:**
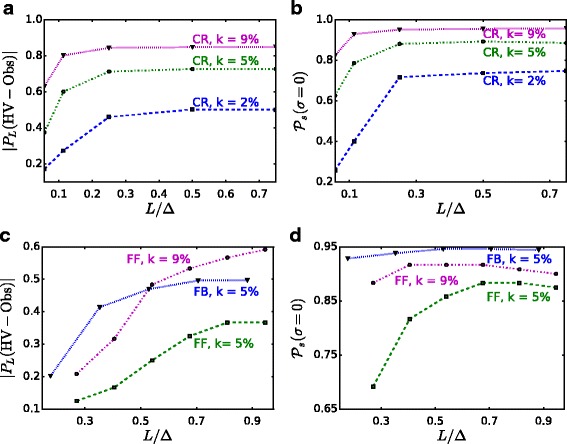
Fraction of nodes in *P*_*L*_(HV-OBS) and success probability in the zero-variance regime ($\mathcal P_{s}(\sigma =0)$) as a function of *L*/*Δ*. **a** CR. **b** CR. **c** F & F, FB. **d** F & F, FB

**Fig. 9:**
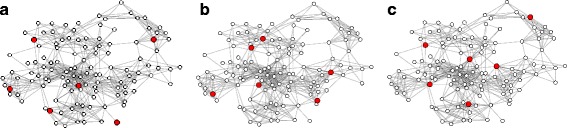
Comparison of the oberver placements LV-OBS, HV-OBS ($L/\tilde {\Delta }=0.5$) and K-MEDIANS with *k*=5*%* on the F & F network. Note the difference between LV-OBS and HV-OBS: LV-OBS contains leafs while HV-OBS has shorter spacing. **a** LV-OBS. **b** HV-OBS ($L/\tilde {\Delta }=0.5$). **c** K-MEDIANS

The cardinality of $P_{L}(\mathcal {O})$ is a good proxy for the performance of $\mathcal {O}$. The value |*P*_*L*_| is increasing in *L* and reaches its maximum for *L* equal to the maximum weighted distance *Δ*. For small *L*, |*P*_*L*_(HV-OBS)|<|*P*_*Δ*_(LV-OBS)| but for *L* large enough this is no longer the case. See Fig. 7a for an example. Our empirical results suggest that *L* should be chosen as the maximum for which |*P*_*L*_(HV-OBS)|≤|*P*_*Δ*_(LV-OBS)|. The key property of HV-OBS with respect to LV-OBS is that observers are spread more *uniformly* without *losing* too much in terms of success probability $\mathcal {P}_{s}$: Fig. 8a shows |*P*_*L*_(HV-OBS)| and $\mathcal {P}_{s}$ as a function of *L*. An a-priori evaluation of the variance threshold above which one should use the HV-OBS placement (and of the appropriate value of the *L* parameter) can be based on the comparison of $\mathcal {P}_{s}$ on a path graph for different values of *L* and *σ* as in Fig. 5a. In fact, looking at Fig. 5 we see that, for small values of $\sigma \mathcal {P}_{s}$ is very close to 1 independently of *L*, hence LV-OBS is the best solution. When *σ* grows, we see that, in order to guarantee an high $\mathcal {P}_{s}$ one must choose smaller and smaller values of *L*. LV-OBS and HV-OBS can give drastically different observers (see Fig. 9a for an example).

## Empirical results

### Datasets

We purposely run our experiments on three very different real-world networks that, in addition to being relevant examples of networks for epidemic spread, display different characteristics in terms of size, diameter, clustering coefficient and average degree (see Table 1), enabling us to test the performance of our methods on various topologies.

**Table 1:** Displays statistics for the networks examined

	|*V*|	|*E*|	min(*w*_*uv*_)	avg(*w*_*uv*_)	max(*w*_*uv*_)	Avg Degree	Diameter	Avg Dist	Avg Clust.
Friends & Families	120	563	4	5.58	7	9.38	6	17.5	0.67
Facebook Messages	1020	6205	1	2.97	5	12.16	5	6.69	0.09
California Roads	1259	1801	1	1.71	9	2.86	66	55.3	0.2

The three networks we consider are:

- Friend & Families (F & F). This is a dataset containing phone calls, SMS exchanges and bluetooth proximity, among a community living in the proximity of a university campus (Aharony et al. 2011). We select the largest connected component of individuals who took part in the experiment during its whole duration. The edges are weighted, according to the number of phone calls, SMSs, and bluetooth contacts.
- Facebook-like Message Exchange (FB) (Opsahl and Panzarasa 2009). As the individuals included in this dataset were living on the same university-campus, the number of messages exchanged is likely to be a good measure of in-person interaction. We selected links on which at least one message was sent in both directions and individuals that had a contact with at least one other individual.
- California Road Network (CR) (California Road Network). In order to obtain a single connected component and remove points that effectively represent the same location, we collapsed the points falling within a distance of 2 km. Moreover we iteratively deleted all leaves. In fact, the roads that cross the state border are not completely tracked in this dataset and terminate with a leaf. Some other leaves might represent remote locations, not necessarily close to the borders, but their influence on the epidemic should anyway be very low.
  The diameter of the CR network is very large compared with that of the other two networks.
  The edges are weighted according to a rescaled version of the real distance (measured in km).

In all three networks, edges are given (non-unit) integer *weights*, which is realistic in many applications as the expected transmission delays are known only up to some level of precision. Integer weights do *not* simplify the localization of the source; in fact, this makes it *more* difficult to distinguish between vertices. For example, if the edges of the CR network were weighted according to the Euclidean distance between the two endpoints, LV-OBS would use only a very small portion of the budget and the comparison with other observer placements would not be meaningful.

### Comparison against benchmarks

We compare LV-OBS and HV-OBS against the following benchmarks:

- ABC (Adaptive Betweenness Centrality): Betweenness Centrality (BC) is a popular method for placing observers for source-localization (see, e.g., (Louni and Subbalakshmi 2014) and (Seo et al. 2012), where it emerges as the best heuristic for observer placement among those tested). It consists of the *k* nodes having the largest BC, which is defined, for all *u*∈*V* as
  $$\text{BC}(u) = \sum_{x, y \in V, x \neq y} \frac{\sigma_{x,y}(u)}{\sigma_{x,y}} $$
  where *σ*_*x*,*y*_ is the number of shortest paths between *x* and *y* and *σ*_*x*,*y*_(*u*) is the number of those paths that passes through *u*. Here we consider an adaptive version of BC (ABC) which iteratively chooses the node that maximizes the betweenness centrality without considering the shortest paths that pass by already-chosen vertices (Yoshida 2014). ABC, with respect to the basic BC, gives less clustered, and hence more efficient, observer sets.
- Coverage-rate (Coverage) (Zhang et al. 2016): This approach maximizes the number of nodes that have an observer as a neighbor, i.e.,
  $$\mathrm{C}(\mathcal{O}) = |\cup_{o \in \mathcal{O}} N_{o}|/n $$
  where *N*_*o*_ denotes the set of neighbors of *o*. It has been shown to outperform several heuristics with a diffusion model and a source-localization setting that are very similar to ours (Zhang et al. 2016).
- k-Median: this is the optimal placement for the closely-related problem of maximizing the detectability of a flow (Berry et al. 2006). The k-Median placement is the set of *k* nodes $\mathcal {O}$ such that
  $$\mathcal{O} = \text{argmin}_{|\mathcal{O}|=k} \sum_{s \in V} (\min_{o \in \mathcal{O}} d(s, o)). $$
  Determining the k-Medians of a network is NP-hard (Kariv and Hakimi 1979), hence we approximate k-Medians with a greedy heuristic.

### Transmission delays

Unless otherwise specified, we sample the transmission delays *X*_*uv*_ from truncated Gaussian random variables with parameters (*w*_*uv*_,*σ* *w*_*uv*_,[^*w*^ *u* *v*/2,^3*w*^ *u* *v*/2]). More precisely, if $Y_{uv} \sim \mathcal {N}(w_{uv}, \sigma w_{uv})$ is a Gaussian random variable, *X*_*uv*_ is obtained by conditioning *Y*_*uv*_ with *Y*_*uv*_∈[^*w*^ *u* *v*/2,^3*w*^ *u* *v*/2]. With respect to the delay distribution assumed by Pinto et al. (Pinto et al. 2012) i.e., ${X_{uv} \sim \mathcal {N}(w_{uv}, \sigma w_{uv})}$, the distribution we assume has the advantage of admitting only strictly positive infection delays. Furthermore, different values of the parameter *σ* result in different regimes for the transmission delays, making our model very versatile. When *σ*=0, we are in the zero-variance regime; when *σ* is large, the distribution of *X*_*uv*_ becomes closer to a uniform random variable *U*([^*w*^ *u* *v*/2,^3*w*^ *u* *v*/2]). Finally, when *σ* is strictly positive but small, ${X_{uv} \approx \mathcal {N}(w_{uv}, (\sigma w_{uv})^{2})}$.

To assess the robustness of our approach for source localization and observer placement, we also experiment with uniformly distributed transmission delays, i.e., for every edge *u* *v*∈*E*, we take *X*_*uv*_∼Unif([(1−*ε*)*w*_*uv*_,(1+*ε*)*w*_*uv*_]). The uniform distribution is, among the unimodal distributions on a bounded support, the one that maximizes the variance (Gray and Odell 1967). Hence, uniform delays are a very challenging setting for source localization.

### Experimental results

We estimate the probability of success $\mathcal {P}_{s}$ and the expected distance $\mathcal {D}$ for different values of the variance parameter *σ*. Our estimations are computed averaging the results obtained choosing each node in turn as the source and generating synthetic epidemics. For the FB and CR datasets, we run 5 simulations per node and value of *σ*; for the F & F dataset, as the network is smaller, we run 20 simulations per node and value of *σ*. For the FB and CR datasets, we localize the source based on the first 20 observations only: Given the large size of these networks, it would be unrealistic to wait for all the nodes to get infected before running the algorithm.

**Fig. 10:**
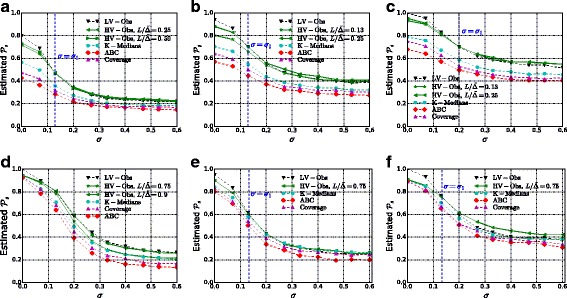
Success probability $\mathcal P_{s}$ as the variance parameter *σ* increases. **a** CR, 2% observers. **b** CR, 5% observers. **c** CR, 9% observers. **d** FB, 5% observers. **e** F & F, 5% observers. **f** F & F, 10% observers

**Fig. 11:**
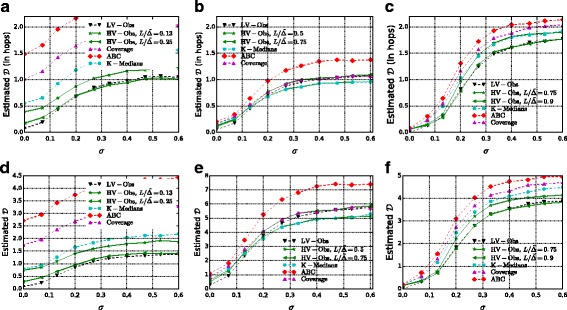
Expected distance $\mathcal {D}=\mathbf {E}[d(s^{\star }, \hat {s})]$ in number of edges (*first row*) and in weighted path length (*second row*) as the variance parameter *σ* increases. **a** CR, 5% observers. **b** F & F, 5% observers. **c** FB, 5% observers. **d** CR, 5% observers. **e** F & F, 5% observers. **f** FB, 5% observers

The results for $\mathcal {P}_{s}$ are displayed in Fig. 10. An approximation of the value *σ*_1_, above which HV-OBS outperforms LV-OBS, is marked with a vertical line. For the expected distance (weighted and in hops), see Fig. 11.

**Fig. 12:**
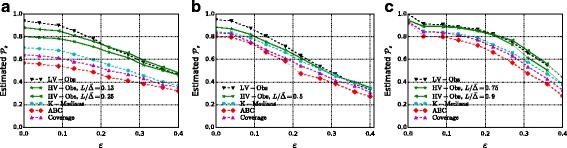
Success probability $\mathcal {P}_{s}$ for uniform transmission delays *X*_*uv*_∼Unif([(1−*ε*)*w*_*uv*_,(1+*ε*)*w*_*uv*_]). **a** CR, 5% observers. **b** F & F, observers. **c** FB, 5% observers

We first take as budget for the observers the minimum budget for which $\mathcal {P}_{s}(\textsc {lv-Obs}) = 1$. This corresponds to *k*∼10*%* for the F & F dataset, *k*∼9*%* for the CR network and *k*∼5*%* for the FB dataset. This is the setting in which we expect the improvement of HV-OBS over LV-OBS to be especially strong: For smaller values of *k* we expect LV-OBS to be nearly optimal even in the high-variance regime because we do not have enough budget to contrast both the topological *undistinguishability* among nodes (what LV-OBS is designed for) and the accumulation of variance (what HV-OBS is designed for).

For the F & F and the CR networks, we also experiment with smaller percentages of observers and consistently find an improvement of HV-OBS over LV-OBS in the high-variance regime: Below a certain amount of variance *σ*_1_ LV-OBS performs better than HV-OBS for any choice of the parameter *L*, whereas above *σ*_1_ a calibrated choice of *L* leads to a significant improvement. Such *L* stays constant for all *σ*>*σ*_1_, i.e., with the notation of Fig. 1 we have *σ*_1_=*σ*_*F*_.

For the FB dataset instead, probably due to the low diameter with respect to the number of nodes, we observe that HV-OBS does not improve on LV-OBS for any value of *L*.

Both LV-OBS and HV-OBS systematically outperform the baseline heuristics for observer placement that we described in “Comparison against benchmarks” section. For the CR dataset the performance of Adaptive Betweenness Centrality is particularly poor. The Coverage Rate heuristic outperforms Adaptive Betweenness Centrality on all three networks (confirming what found by by Zhang et al. (2016)) but is consistently less effective than K-Medians and than our methods.

Finally in Fig. 12, we consider uniform transmission delays, and we measure whether, without making any changes, our observer placement still performs well. We find comparable results which suggest that our observer placement is not dependant on the exact transmission model and that the variance of the transmission delays is really a key factor for a good observer placement.

## Related work

The problem of source localization has been widely studied in recent years, we survey the works that are more relevant to ours and refer the reader to the survey by Jiang et al. (2014) for a more complete review of the different approaches.

**Transmission delays.** Many transmission models for epidemics have been studied (Lelarge 2009) and considered for source localization. Although discrete-time transmission delays are common (Luo et al. 2014; Prakash et al. 2012; Altarelli et al. 2014), in order to better approximate realistic settings, much work (including ours) adopt continuous-time models with varying distributions for the transmission delays; e.g., exponential (Shah and Zaman 2011; Luo and Tay 2012) or Gaussian (Pinto et al. 2012; Louni and Subbalakshmi 2014; Louni et al. 2015; Zhang et al. 2016). In the same line of the latter class of works, we use *truncated* Gaussian variables, which gives us the advantage of ensuring that infection delays are strictly positive.

**Source localization.** Many approaches (Zheng and Tan 2015; Prakash et al. 2012; Sundareisan et al. 2015), beginning with the seminal work by Shah and Zaman Shah and Zaman (2011), rely on knowing the state of the *entire* network at a fixed point in time *t*; this is often called a *complete observation* of the epidemic. These models use maximum likelihood estimation (MLE) to estimate the source. The results of (Shah and Zaman 2011) have been extended in many ways, for example in the case of multiple sources (Luo and Tay 2012) or to obtain a *local* source estimator (Dong et al. 2013). An alternate line of work considers a complete observation of the epidemic, except that the observed states are *noisy*, i.e., potentially inaccurate (Zhu and Ying 2013; Sundareisan et al. 2015). As assuming the knowledge of the state of all the nodes is often not realistic, *partial observation* settings have also been studied. In such a setting, only a subset of nodes $\mathcal {O}$ reveal their state. In this line of work, the observers are mainly *given*, either arbitrarily or via a random process, and the problem of *selecting* observers is not addressed. For example, when a fraction *x* of nodes are randomly selected, Lokhov et al. (2014) propose an approach which relies on the knowledge of the state (S, I or R) of a fraction of the nodes in the graph at a given moment in time and in which the starting time of the epidemic, if unknown, can be inferred from the data available. When the nodes are independently selected to be observers, an approach to source estimation based on the notion of *Jordan center* was proposed (Luo et al. 2014) and has since been used for source estimation, especially with regard to a *game theoretic* version of epidemics (Fanti et al. 2015). This line of work does not assume infection times are known, which we believe is, in many cases, an unnecessary limitation. Indeed by using infection times we can achieve exact source localization in the zero-variance setting with sufficiently many observers (Chen et al. 2014), whereas this is not true otherwise.

**Observer placement.** Natural heuristics for observer placement (e.g., using high-degree vertices or optimizing for distance centrality) were first evaluated under the additional assumption that infected nodes know which neighbor infected them (Pinto et al. 2012). Later, Louni and Subbalakshmi (2014) proposed, for a similar model, to place the observers using a Betweenness-Centrality criterion (which we use as a benchmark, see “Comparison against benchmarks” section), and extended it to noisy observations (Louni et al. 2015). These and other heuristic approaches for observer placement are evaluated empirically by Seo et al. (2012); they reach the conclusion that, among the placements they evaluate, the Betweenness-Centrality criterion performs the best. In their work the source is estimated by ranking candidates according to their distance to the set of observers, without using the time at which the observers became infected. Once again, this approach is inherently limited by the fact that it does not make use of the time of infection.

The problem of *minimizing* the number of observers required to detect the precise source (as opposed to *maximizing* the performance given a *budget* of observers) has been considered in the zero-variance setting. For trees, given the time at which the epidemic starts, the minimization problem was solved by Zejnilovic et al. (2013). Without assuming a tree topology and a known starting time, approximation algorithms have been developed towards this end (Chen et al. 2014) (still in a zero-variance setting). However, in a network of size *n*, the number of observers required, even if minimized, can be up to *n*−1, hence, a budgeted setting is practically more interesting. For trees, the budgeted placement of observers was solved by using techniques different from ours (Celis et al. 2015). However these techniques heavily rely on the tree structure of the network and do not seem to be extendible to other topologies. In a recent work, Zhang et al. (2016) consider selecting a fixed number of observers using several heuristics such as Betweenness-Centrality, Degree-Centrality and Closeness-Centrality and they show that none of these methods are satisfactory. They introduce a new heuristic for the choice of observers, called *Coverage-Rate*, which is linked to the total number of nodes neighboring observers, and show that an approximated optimization of this metric yields better performance. Connecting the budgeted placement problem to the un-budgeted minimization problem, we provably outperform their approach in low-variance settings. For example, in the low-variance setting, on cycles of odd-length *d* with budget *k*=2, any two nodes at distance more than 2 are equivalent with respect to Coverage-Rate, but they maximize $\mathcal {P}_{s}$ only if they are at distance (*d*−1)/2; our approach instead, selects this optimal placement. Moreover, the effect of the variance in the transmission delays is neglected by Zhang et al., leaving open the question of whether their approach works in general. We consider Coverage-Rate as one of our baselines.

## Conclusion and future work

In this work, we have taken a principled approach towards budgeted observer placement for source localization, which shows a dichotomy between the low and high-variance regimes. We developed complementary approaches to handle both regimes. We evaluated our approaches against state-of-the-art and alternative heuristics showing a better performance of the algorithms proposed in this paper.

A direction for future work would be to measure the performance with *worst case* rather than *average case* metrics: if we can handle (adversarially chosen) source distributions where the epidemic starts at the least-observed location, then this gives a bound on the performance with an *arbitrary prior distribution*.

A natural extension of our model was recently studied in a work by Spinelli et al. (2017) which accounts for two stages of observation. In the first stage, as in this work, a small set of observers are selected to monitor the network. In the next stage, once an epidemic begins, additional observers are deployed in the relevant region of the network to localize the source. The latter work does not address interesting questions such as the impact of the initial budget deployed and of the position of the observers chosen in the first stage. The techniques and the results of this paper pave the way for answering these questions which we consider of high practical importance.

## Endnotes

^1^ A preliminary version of this work was presented at the 54^*th*^ Annual Allerton Conference on Communication, Control, and Computing (Spinelli et al. 2016).

^2^ Note that in Figs. 2 and 5a we compute the value of the success probability $\mathcal {P}_{s}$ assuming Gaussian distributed delays (and ignoring that, with low probability, negative delays could appear) because this is the only distribution that makes the exact computation of this value feasible. However, in all experiments we only consider non-negative distributions for *X*_*uv*_.

^3^ See “Metrics for source localization” section for a discussion of alternative metrics for source localization.

^4^ Call $\mathcal {O}_{opt}$ the optimal observer placement for any of the metrics considered and $\mathcal {L}$ the leaves set. If $\mathcal {O}_{opt} \nsubseteq \mathcal L$ there would be observer $o\in \mathcal {O}_{opt}$ equivalent to a leaf $\ell \notin \mathcal {O}_{opt}$ and by substituting *o* with *ℓ* we would break [ *o*] in two or more smaller equivalence classes. In this way the value of the metric considered would get closer to its optimum.

^5^ The standard error of measurement is not reported for the sake of readability but it was checked to be small.

^6^ Lyapunov condition with *δ*=1 is easily verified for a sequence of independent and uniformly bounded random variables (see Example 27.4 in (Billingsley 1995) for more details).

^7^ https://github.com/bmspinelli/observers_for_source_loc.

## Appendix A: Double Resolving Sets

The problem of *minimizing* the required number of observers in order to perfectly identify the source in the zero-variance setting has been studied (Chen et al. 2014); an observer set $\mathcal {O}$ such that $\mathcal {P}_{s}(\mathcal {O}) = 1$ is called a Double Resolving Set (DRS). While the original formulation of the DRS problem is slightly different, this version follows straightforwardly from our observations in “The low-variance regime” section.

### **Definition 3**

*(Double Resolving Set)* Given a network $\mathcal {G}$, *S*⊆*V* is said to be a Double Resolving Set of $\mathcal {G}$ if for any *x*,*y*∈*V* there exist *u*,*v*∈*S* s.t. *d*(*x*,*u*)−*d*(*x*,*v*)≠*d*(*y*,*u*)−*d*(*y*,*v*).

Finding a Double Resolving Set of minimum size is known to be NP-hard (Kratica et al. 2009). An approximation algorithm, based on a greedy minimization of an *entropy* function, has been studied. Note that this has no connection to true information-theoretic entropy.

### **Definition 4**

*(Entropy (* Chen et al. 2014 *))* Let $\mathcal {G}$ a network, $\mathcal {O} \subseteq V$, $|\mathcal {O}|=k$ a set of observers. The entropy of $\mathcal {O}$ is

$$H_{\mathcal{O}} = \log_{2}\left(\prod_{[u]_{\mathcal{O}} \subseteq V}|[u]_{\mathcal{O}}|!\right) $$

Note that $H_{\mathcal {O}}$ is minimized if and only if each equivalence class consists of only one node and hence if and only if $\mathcal {P}_{s}=1$. However, despite the fact that $H_{\mathcal {O}}$ is minimized when $\mathcal {P}_{s}$ is maximized and that both act on the same set of equivalence classes for a given $\mathcal {O}$, the greedy processes that minimize $H_{\mathcal {O}}$ and maximize $\mathcal {P}_{s}$ are not the same. This can be seen by rewriting both objective functions in the following way. Let [*c*_1_,…,*c*_*q*_] be the sequence of equivalence class sizes. Then $H_{\mathcal {O}}$ can be written as $H_{\mathcal {O}}([c_{1},.., c_{q}]) = \sum _{i=1}^{l} \sum _{j=2}^{c_{i}} \log (j) = \sum _{i=2}^{\max {c_{j}}} \log (i) \#\{c_{j} \geq i\}.$ Analogously we have the following equality for the success probability $\mathcal {P}_{s}([c_{1}, \ldots, c_{q}])$: $n (1-\mathcal {P}_{s}([c_{1}, \ldots, c_{q}])) = n - q = \sum _{i=2}^{\max {c_{j}}} \#\{c_{j} \geq i\}$

Hence, though similar in spirit, a greedy minimization of $H_{\mathcal {O}}$ is not related to a greedy optimization of $\mathcal P_{s}$ (or $\mathbf {E}[d(s^{*}, \hat s)]$).

## Appendix B: Hardness of Budgeted Observer Placement

### **Theorem 3**

Given a network $\mathcal {G}=(V,E)$ and a budget *k*, finding an observer set $\mathcal {O}$ which maximizes $\mathcal {P}_{s}$ is NP-hard.

### *Proof*

We will prove that the budgeted observer placement is NP-hard with a reduction from the DRS problem (see Appendix A: Double Resolving Sets section), i.e., given a polynomial-time algorithm for the budgeted observer placement problem, we will prove that we can solve the DRS problem in polynomial time.

Assume that we have a polynomial-time algorithm that takes as input a network $\mathcal {G} = (V,E)$ and a budget *k*, and outputs a set $\mathcal {O} \subseteq V$ of size *k* such that $\mathcal {P}_{s}$ is maximized. Recall from “The low-variance regime” section that given a network $\mathcal {G}$ and a set $\mathcal {O}$, the probability $\mathcal {P}_{s}$ can be calculated in time *O*(*n*) where *n*=|*V*| (it is enough to compute the *n* distances vector with respect to $\mathcal {O}$ and any reference observer $o_{1}\in \mathcal {O}$). Hence, we will construct an algorithm for the DRS problem.


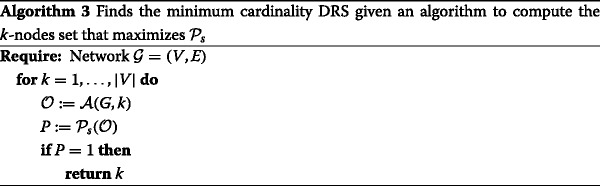


Since the full set *V* always resolves the network, the program is well defined (i.e., it always returns *some* *k*). Moreover, it returns precisely the minimum budget *k* required in order to attain $\mathcal {P}_{s} = 1$. Lastly, it is clear that the runtime is at most *O*(*n*(*p*(*n*)+*n*)) where *p*(*n*) is the running time of algorithm. Hence, we have a polynomial-time algorithm for the DRS problem. □

## Appendix C: Alternative objective functions for Algorithm 1

We present the results of the experiment described in Comparison with benchmarks section. Let us here denote LV-OBS with *Φ* for consistency of notation.

Table 2 compares LV-OBS, *Φ*_*ent*_ and *Φ*_*dist*_, for different topologies and different budgets *k*, in terms of both $\mathcal {P}_{s}$ and $\mathcal {D}$. The results are given in the form of (averaged) relative differences.^5^

**Table 2:** Comparison of LV-OBS (*Φ*) with the greedy algorithms that minimize the entropy function of (Chen et al. 2014) (*Φ*_*ent*_) and the expected distance (*Φ*_*dist*_)

	$\rho (\mathcal {P}_{s}, \Phi, \Phi _{dist})$	$\rho (\mathcal {D}, \Phi _{dist}, \Phi)$	$\rho ({\mathcal P_{s}}, \Phi, \Phi _{ent})$
Random Geometric Network, *N*=100, *r*=0.2			
*k*=2	-0.205	0.101	-0.033
*k*=4	-0.014	-0.003	-0.007
*k*=8	-0.003	-0.002	-0.003
Barabàsi Albert Network, *N*=100, *m*=3			
*k*=2	-0.168	0.023	-0.037
*k*=4	-0.039	0.025	-0.028
*k*=8	-0.004	-0.003	0.005

We denote the relative difference of *x* and *y* with respect to *f* as

$$\rho(f, x, y)\stackrel{\text{def}}{=} \frac{f(y) - f(x)}{f(x)}. $$

Since the expected distance can be equal to 0 we add 1 to the denominator when comparing values of $\mathcal {D}$, i.e.,

$$\rho(\mathcal{D}, x, y)\stackrel{\text{def}}{=} \frac{\mathcal{D}(y) - \mathcal{D}(x)}{\mathcal{D}(x) + 1}. $$

## Appendix D: Source Localization in the High-Variance Regime

We describe here how we compute the estimated source $\hat {s}$ in the high-variance regime. Denote by $T_{\mathcal {O}}$ the vector of the observed infection times. If the transmission delays are Gaussian-distributed, $\mathcal {G}$ is a tree, the maximum likelihood (ML) estimator defined as

$$\hat{s} \in \arg \max_{\substack{s\in V}} \mathbf{P}(s|T_{\mathcal{O}}), $$

has a tractable closed form (Pinto et al. 2012). Note that the model of (Pinto et al. 2012) additionally assumed infected observers knew the neighbor that infected them; this assumption is not essential for the derivation of the ML estimator and it is not required in our work.

In particular, given a set of observers

$$\mathcal{O}=\{o_{1}, o_{2}, \ldots, o_{k}\} \subseteq V, $$

the vector of the observed infection delays $\tau = [t_{2}-t_{1}, \ldots, t_{k}-t_{1}] \in \mathbb {R}^{k-1}$ is distributed as $\mathcal {N}(\mathbf {d}_{s, o_{1}}, \mathbf {\Lambda }_{\mathcal {O}})$ where $\mathbf {d}_{s, o_{1}}$ is the distance vector of Definition 2 and the covariance matrix $\mathbf {\Lambda }_{o_{1}}$ is

8 $$ \boldsymbol\Lambda_{o_{1}, (k,i)}=\sigma^{2} \left\{\begin{array}{ll} \sum_{(u,v) \in \mathcal{P}(o_{1},o_{k+1})} w_{uv}^{2} & k=i\\ \sum_{(u,v) \in \mathcal{P}(o_{1},o_{k+1})\cap \mathcal{P}(o_{1},o_{i+1})} w_{uv}^{2}& k \neq i, \end{array}\right.  $$

with $\mathcal {P}(x,y)$ denoting the set of edges in the unique path between *x* and *y*. Hence the ML estimator is

9 $$ \begin{array}{ll} \hat{s}&\in\arg \max_{\substack{s\in V}}\frac{\exp \left(-\frac{1}{2}\left(\tau-\mathbf{d}_{s,o_{1}}\right)^{\top}{\mathbf{\Lambda}_{o_{1}}}{^{-1}} \left(\tau-\mathbf{d}_{s, o_{1}}\right)\right)}{|\mathbf{\Lambda}_{o_{1}}|^{1/2}}\\ &=\arg \max_{\substack{s\in V}} \Big[\mathbf{d}_{s}^{\top}{\mathbf{\Lambda}_{o_{1}}}^{-1} \left(\tau-\frac{1}{2}\mathbf{d}_{s,o_{1}}\right)\Big]. \end{array}  $$

On non-tree networks, the multiplicity of paths linking any two nodes makes source estimation more challenging. As claimed in (Pinto et al. 2012), the same estimator can be used as an approximation of the ML estimator for a non-tree network by assuming that the diffusion happens only through a BFS (*Breadth-First-Search*) tree rooted at the (unknown) source. In this case the paths which appear in the definition of the covariance matrix $\mathbf {\Lambda }_{o_{1}}$ are computed on the BFS tree rooted at the candidate source considered. Hence $\mathbf {\Lambda }_{o_{1}}$ depends on the candidate source and the ML estimator is

10 $$ \hat{s}_{\text{BFS}} \in\arg \max_{\substack{s\in V}}\frac{\exp \Big(-\frac{1}{2}(\tau-\mathbf{d}_{s, o_{1}})^{\top}{\mathbf{\Lambda}^{s}_{o_{1}}}{^{-1}} (\tau-\mathbf{d}_{s,o_{1}})\Big)}{\Big|\mathbf{\Lambda}^{s}_{o_{1}}\Big|^{1/2}}.  $$

In this work, we adopt (10) as the source estimator in the noisy case. In fact, even if our edge delays are not Gaussian-distributed, under the hypothesis of sparse observations, we can apply the Central Limit Theorem (CLT) to approximate the sum of the edge delays with Gaussian random variables: if all edges have the same weight we can apply the CLT for i.i.d. random variables; if this is not the case, we can apply Lyapunov’s version of CLT.^6^Using (10) to compute the ML estimator, the likelihood of nodes in the same equivalence class can result to be different as an artefact of the BFS-tree approximation. Hence, for consistency with our source-localization method in the low-variance case, we compute an average likelihood and estimate that the source is in the class with the higher average likelihood. Then, once an equivalence class for the source is estimated, we select $\hat {s}$ by sampling the prior probability on the position of the source (if available) or by uniform sampling from the estimated equivalence class.
